# Bronchial hygiene techniques in patients on mechanical ventilation: what are used and why?

**DOI:** 10.1590/S1679-45082018AO3856

**Published:** 2018-04-06

**Authors:** Isabela Naiara Evangelista Matilde, Raquel Afonso Caserta Eid, Andréia Ferreira Nunes, Alexandre Ricardo Pepe Ambrozin, Renata Henn Moura, Denise Carnieli-Cazati, Karina Tavares Timenetsky

**Affiliations:** 1 Hospital Israelita Albert Einstein, São Paulo, SP, Brazil; 2 Universidade Estadual Paulista “Júlio de Mesquita Filho”, Marília, SP, Brazil

**Keywords:** Respiration, artificial, Physical therapy modalities, Physical therapy department, hospital, Evidence-based medicine, Respiração artificial, Modalidades de fisioterapia, Serviço hospitalar de fisioterapia, Medicina baseada em evidências

## Abstract

**Objective:**

To analyze and describe the maneuvers most commonly used in clinical practice by physical therapists and the reasons for choosing them.

**Methods:**

A prospective multicenter study using a questionnaire. The sample consisted of physical therapists from five hospitals (three private hospitals, a teaching hospital and a public hospital).

**Results:**

A total of 185 questionnaires were filled in. Most professionals had graduated 6 to 10 years before and over had over 10 years of intensive care unit experience. The most often used maneuvers were vibrocompression, hyperinflation, postural drainage, tracheal suction and motor mobilization. The most frequent reason for choosing these maneuvers was “I notice they are more efficient in clinical practice.”

**Conclusion:**

Physical therapy is mostly based on individual experience acquired in the clinical practice, and not on the scientific literature.

## RESUMO

**Objetivo:**

Analisar e descrever as manobras mais usadas na prática clínica pelos fisioterapeutas e os motivos para esta escolha.

**Métodos:**

Estudo prospectivo e multicêntrico, realizado por meio de um questionário. A amostra foi composta por colaboradores fisioterapeutas de cinco hospitais, sendo três particulares, um hospital escola e um público.

**Resultados:**

Foram preenchidos 185 questionários. A maioria dos profissionais possuía de 6 a 10 anos de formação e mais de 10 anos de experiência em unidades de terapia intensiva. As manobras mais assinaladas foram: vibrocompressão, hiperinsuflação, drenagem postural, aspiração traqueal e mobilização motora. O motivo de escolha prevalente destas manobras foi “Eu vejo ser mais eficaz na prática clínica”.

**Conclusão:**

A fisioterapia baseia-se na prática clínica adquirida ao longo da experiência individual, não sendo fomentada pela literatura científica.

## INTRODUCTION

In most hospitals, physical therapy is seen as part of patients’ treatment in intensive care units (ICU).^(^ ^1^ ^)^ Although techniques for bronchial hygiene are routinely carried out in ICU patients, several studies evaluating their efficacy have found heterogeneous results and their effectiveness has remained unproven. The application of vibrocompression in mechanically ventilated patients improves peripheral oxygen saturation.^(^ ^2^ ^)^ Thirty minutes after a session of vibrocompression and increased expiratory flow, blood pressure drops; however, there is no significant change in the volume of secretion removed by either technique.^(^ ^3^ ^)^ In contrast, the respiratory physical therapy protocol is effective in reducing airway resistance as compared to tracheal suction, and this decrease is sustained for 2 hours after using the protocol. The same does not occur if only tracheal suction is conducted.^(^ ^4^ ^)^

A review of 7 studies, with a total of 126 patients with bronchiectasis and chronic obstructive pulmonary disease, treated with various bronchial hygiene techniques, such as postural drainage, percussion, vibration, directed cough and the forced expiratory technique, found that none of them had significant effect on pulmonary function, achieving only bronchial hygiene.^(^ ^5^ ^)^ Manually assisted cough can change the respiratory system mechanics, *i.e* the resistive forces and displaces airways secretion.^(^ ^6^ ^)^

When comparing manual hyperinflation in patients on mechanical ventilation (MV) to isolated tracheal suction, there was a 30% increase in dynamic compliance after the first technique, as well a greater volume of secretion removed.^(^ ^7^ ^)^ On the other hand, there are also records of non-significant variation when comparing manual techniques applied on the chest with tracheal suction, also in MV patient.^(^ ^8^ ^)^

A study conducted in 2004 suggested that chest compression prior to tracheal suction does not improve airway clearance, oxygenation, or ventilation in patients on MV.^(^ ^9^ ^)^ However, stacking, chest compression and the association of these two techniques were effective in increasing peak cough flow, therefore reproducing cough.^(^ ^10^ ^)^ Physical therapy in patients on MV significantly decreased the clinical scores of lung infection and mortality rates in the study group as compared to controls.^(^ ^11^ ^)^

Considering bronchial hygiene techniques are widely used in several ICUs despite the discrepancy found in the literature regarding their effectiveness, we understand that some physical therapists working in this area have their preferences regarding the technique to be used.

## OBJECTIVE

To analyze and describe the maneuvers most used by physical therapists in clinical practice and the reasons for their choices.

## METHODS

A prospective multicenter study carried out using a specific questionnaire (Annex 1). The sample consisted of physical therapists from five hospitals - in that, two public and three private organizations. Only one of the five hospitals is located in the countryside of the State of São Paulo; the others are in the state capital city. The project was approved by the Internal Review Board, under number 918.955, CAAE: 35478014.0.0000.0071; afterwards, the printed questionnaires and Informed Consent Forms were sent to the head of each organization, who passed them on to their employees. The practitioners answered and forwarded the questionnaire to the person in charge of the research in their institution, who further sent them to the research coordinator.

We included practitioners working at-will employment relationship or on-call system, with specialization in hospital physical therapy and/or intensive care therapy, and assisting adult patients on MV were included. The professionals who did not return the questionnaire to the researchers were excluded from the study. The responders’ names were kept confidential, and only the results of questionnaires were disclosed.

Regardless of the hospital, the physical therapists included in the study had a workload of 30 hours per week, in which they had to see patients with different demands (5 to 15 patients during a 6-hour shift). The sessions were heterogeneous, ranging from 20 to 50 minutes on average, and included analysis of laboratory and imaging tests, re-expansion or bronchial hygiene maneuvers, motor physical therapy and bureaucratic routines.

The questionnaire contained questions about the bronchial hygiene maneuvers commonly used by physiotherapists in their clinical practice in patients on MV and the reason for choosing them. Participants could indicate more than one maneuver or reason for using them. These data served as foundation to understand the situation of each organization regarding the choice of bronchial hygiene techniques as treatment, and their relation with the number of patients seen per shift. It also provided data on the number of years of experience and time lapse since graduation in physical therapy. The reasons for choosing the maneuvers were also considered. Information regarding the participant sex and age was collected.

### Statistical analysis

An exploratory descriptive analysis of all variables was performed to characterize the practitioners who answered the questionnaire, describe the main techniques used and the reason for their choice. Qualitative variables were described using absolute and relative frequencies (percentages). Quantitative variables were expressed as means, medians, standard deviations, minimum and maximum values.

The characteristics of interest were analyzed in relation to the types of maneuvers performed and reasons for choosing them, and the χ^2^ test, Fisher's exact test or likelihood ratio was used to verify the association among them. The level of significance was set at 0.05. We used the Statistical Package of Social Science (SPSS), version 20.0.

## RESULTS

Practitioners from five hospitals completed 185 questionnaires. Age ranged from 22 to 47 (±5) years. Table 1 depicts the characteristics of the population included in the study. The majority of professionals had graduated in physiotherapy 6 to 10 years before the study (43.2%), followed by 31.9% who reported having graduated more than 10 years before. Regarding the time of experience in ICU, most had 6 to 10 years (42.2%). In respect to the number of patients seen per shift duty, 41.1% said they handled 4 to 6 patients per 6-hour shift, followed by 31.4% who handled 9 to 10 patients.

**Table 1:** Characteristics of the population

Hospital (n)		
	1	78
	2	35
	3	43
	4	9
	5	20
Sex (%)		
	Female	79.5
	Male	20.5
Age, mean		31.5 (±5)
Time since graduation, years (%)		
	≤2	4.4
	3-5	20.5
	6-10	43.2
	>10	31.9
Experience in ICU, years (%)		
	≤2	18.4
	3-5	20.5
	6-10	42.2
	>10	18.9
Patients seen in each shift (%)		
	4-6	41.1
	7-8	3.2
	9-10	31.4
	11-12	13.5
	13-15	5.9
	>16	4.9

ICU: intensive care unit.

Table 2 describes the specific characteristics of each hospital, in relation to most alternatives chosen in each organization. The maneuvers most often mentioned by the responders were vibrocompression, hyperinflation, postural drainage, tracheal suction and motor mobilization. The alternative predominantly chosen as the reason for using the maneuvers was: “I notice they are more efficient in clinical practice”. Chart 1 shows the maneuvers and reasons most frequently chosen in each hospital.

**Table 2:** Characteristics observed at hospitals

Hospital	Patients (years)	Experience (years)	Time working at the ICU (years)
1	4-6, n=62	6-10, n=37	>11, n=34
2	9-10, n=11	6-10, n=15	6-10, n=18
3	4-6, n=42	6-10, n=18	>11, n=23
4	9-10, n=9	6-10, n=4	6-10, n=4 >11, n=4
5	9-10, n=20	6-10, n=10	>11, n=9

ICU: intensive care unit.

**Chart 1:** Main maneuvers and reasons observed in the participating hospitals

Hospital	Bronchial hygiene maneuvers	Reason
1	Videocompression (n=65)	I notice they are more efficient in clinical practice (n=71)
	Postural drainage (n=63)	
	Hyperinflation (n=68)	
	Tracheal suction (n=74)	
	Motor mobilization (n=60)	
2	Videocompression (n=21)	I notice they are more efficient in clinical practice (n=31)
	Hyperinflation (n=20)	
	Suction (n=34)	
	Motor mobilization (n=32)	
3	Videocompression (n=35)	I notice they are more efficient in clinical practice (n=36)
	Hyperinflation (n=43)	The literature shows it is more efficient (n=30)
	Tracheal suction (n=38)	
	Motor mobilization (n=34)	
4	Videocompression (n=7)	I notice they are more efficient in clinical practice (n=9)
	Postural drainage (n=6)	
	Hyperinflation (n=8)	
	Tracheal suction (n=9)	
	Motor mobilization (n=9)	
5	Videocompression (n=14)	I notice they are more efficient in clinical practice (n=19)
	Hyperinflation (n=18)	
	Tracheal suction (n=19)	

When the maneuvers were correlated with the number of years since graduation, there was significant variation in the choice for percussion (p=0.028), compression (p=0.034) and postural drainage (p=0.006), indicating that practitioners who had longer time interval since graduation tended to use more these maneuvers. The length of experience in ICU was found significant in relation to the percussion maneuver (p=0.012) and postural drainage (p=0.029), suggesting that, as the professionals acquired more experience in intensive care, these techniques were more often chosen as a treatment strategy in clinical practice.

When correlating the reason for choosing the maneuvers with the number of years since graduation in physiotherapy and years of experience in ICU, significance was found in the option “I notice they are more efficient in clinical practice”, that is, professionals with more years since graduation and longer experience chose bronchial hygiene maneuvers based on clinical practice.

Correlating the number of patients treated per shift to the maneuvers, the more patients the professionals had to see per period, the less the use of postural drainage and motor mobilization (p=0.001 and 0.01, respectively). In our sample we found that the older the age group of professionals who answered the questionnaire, the more the following maneuvers were chosen: vibration (p=0.005), percussion (p=0.009), postural drainage (p=0.009), endotracheal suction (p=0.003), and motor mobilization (p=0.009).

## DISCUSSION

Bronchial hygiene maneuvers are resources widely used by physical therapists in intensive care with the objective of assisting mucociliary clearance and preventing complications due to accumulated secretions in the airways. Although they are routinely used, the literature is heterogeneous in regard to their true efficacy. Some studies show benefits, while others demonstrate no difference regarding their effects, as well as limitations in the tools used to evaluate the techniques applied and their clinical reproducibility, what is a barrier to the development of a reliable database for all areas of respiratory physiotherapy.^(^ ^12^ ^)^

Among the hospitals analyzed, the main reason for choosing the maneuvers was “I notice they are more efficient in clinical practice”, indicating the lack of credibility or sufficient resources in the literature to support evidence-based medicine. A systematic review carried out in 2013^(^ ^13^ ^)^ stated the studies analyzing the effect of non-pharmacological techniques of airway clearance had small samples and significant variation on the type of population studied, as well as few benefits regarding gas exchange and duration of MV. The review also concluded that further studies were needed to obtain a real picture of the benefits and disadvantages of using bronchial hygiene maneuvers. It is worth recalling the difficulty in obtaining homogeneity of patients included in the studies, due to great diversity of diseases found in general ICUs, in addition to the common barriers that all studies with critical patients present, such as hemodynamic instability, neurological conditions and not obtaining consent from the family. Moreover, there is a possible inter-researcher variation, *i.e* or vibrations are applied with the same intensity and frequency in all patients evaluated, what may lead to variations in the results obtained.^(^ ^14^ ^)^

Physiotherapeutic intervention through bronchial hygiene maneuvers improves the rheological profile of the mucus, moving it more easily.^(^ ^15^ ^,^ ^16^ ^)^ In this study all physiotherapists pointed out at least one maneuver as being commonly used in their daily routine in the ICU, and the most often chosen were vibrocompression, manual hyperinflation, postural drainage, suction and motor mobilization. The choice of the ideal maneuver when seeing patients depends on patient's age and severity of disease, ease of use, compliance to the treatment plan in view of the pathophysiology, and patient comfort or collaboration.^(^ ^17^ ^)^ Several studies^(^ ^18^ ^,^ ^19^ ^)^ identified how respiratory and motor physical therapies impact in reducing hospital costs and mortality, but further research is needed to justify such an impact, since it is multifactorial.

The postural drainage maneuver demands a specific time to be performed, since it requires the action of gravity for secretion flow.^(^ ^20^ ^)^ In our study, the number of patients treated per shift correlates with carrying out this maneuver, *i.e* is used. This result can be justified by the shortage of time seen in services where the physiotherapist must care for a greater number of patients and the need for time dedicated to postural drainage, rendering this maneuver difficult in these cases. A similar result found in our study is related to motor mobilization, as a consequent form of secretion movement, and the use of this technique is reduced as the professionals need to carry out a greater number of visits.

The present study pointed that postural drainage, compression and percussion are more used by physical therapists with longer experience in ICU and more years since graduation in physiotherapy; however, the maneuvers most often pointed out by all physiotherapists were vibrocompression, manual hyperinflation, suction and motor mobilization. Studies in bronchial hygiene maneuvers are contradictory, scenario seen since the mid-1980's,^(^ ^21^ ^–^ ^23^ ^)^ when research including percussion maneuvers, postural drainage, vibration, and assisted cough began to be studied. Recent studies have shown that the maneuvers of vibrocompression, manual hyperinflation and tracheal suction tend to be more highlighted in the literature.^(^ ^2^ ^,^ ^4^ ^,^ ^8^ ^,^ ^9^ ^,^ ^24^ ^)^

Clinical reasoning is a decision-making process based on clinical evaluation, and it allows for selection of a more appropriate intervention for treatment.^(^ ^25^ ^)^ Likewise other health professions, clinical reasoning in physical therapy involves cognitive processes, such as pattern recognition, as well as deductive aspects to solve clinical problems.^(^ ^26^ ^)^ Professional experience in physiotherapy brings in greater expertise in problem solving and, as a consequence, adjusting the choice of techniques best tuned and more efficient as observed in clinical practice, base on previously described theory.^(^ ^27^ ^)^ The absolute majority of participants in our sample pointed clinical practice as the reason for choosing certain maneuvers, suggesting that the choice and the reasons for doing so are based primarily on the individual professional experience, and do not corroborate evidence-based medicine.^(^ ^28^ ^)^

Hypothetical-deductive reasoning remains the most enduring clinical model in medicine. In this model, clinicians acquire initial information on patients and, from these clues, hypotheses are raised and although being cognitive, they are based on scientific research, in which experimentation produces a practical result.^(^ ^29^ ^)^ Other form of clinical reasoning encompasses pattern recognition, which, in turn, is based on information previously stored by the professional. These two ways of reasoning are used at different times; the former is commonly recognized in inexperienced professionals or experts confronted by an unknown problem; the second, in people more experienced in their domain.^(^ ^30^ ^)^

### Study limitations

We did not analyze separately professionals that held more titles than specialization, and this might have interfered in the discussion of results. We suggest that a future study should take these specifications into account. The objective of the study was not to evaluate each hospital separately or their management, but to assess the responses given by the group of participating physiotherapists. The difference of the sample found in each hospital and, mainly, the variation in size of the team at public and private hospitals hinder a specific evaluation of each organization.

## CONCLUSION

The most often used bronchial hygiene maneuvers at bedside by physical therapists were vibrocompression, hyperinflation, postural drainage, tracheal suction and motor mobilization. The most cited reason for choosing them was “I notice they are more efficient in clinical practice.” Most of the study participants based themselves on clinical practice gained through individual experience, not by the scientific literature. Further national studies are needed to ensure the real benefits and drawbacks in performing bronchial hygiene maneuvers.

## Annex 1 Questionnaire provided to the physical therapists who participated in the study

Hospital____________________________________________________________________________________________________

Physical therapist:____________________________________________________________________________________________________ Sex: M (_)_F (_) Age: ___________ How many years since graduation?

(_) Up to 2 years
(_) 3 to 5 years
(_) 6 to 10 years
(_) More than 11 years

How many years of experience in ICU?

(_) Up to 2 years
(_) 3 to 5 years
(_) 6 to 10 years
(_) More than 11 years

How many patients you see, in average, per working shift?

(_) 4 to 6
(_) 7 to 8
(_) 9 to 10
(_) 11 to 12
(_) 13 to 15
(_) More than 16

Which maneuvers do you use on your daily practice for patients on mechanical ventilation?

(_) Vibration
(_) Compression
(_) Vibrocompression
(_) Percussion
(_) Manual hyperinflation (AMBU)
( ) Postural drainage
(_) Acceleration flow
(_) Endotracheal suction
(_) Motor mobilization
(_) I don't usually do them
(_) Other. Which? __________________________________________________

Why do you use these maneuvers?

(_) The literature proves they are efficient
(_) I notice they are more efficient in clinical practice
(_) It is faster
(_) Patients report improvement
(_) I find it easier
(_) Other. Which? __________________________________________________
